# Association Between Red Cell Distribution Width and In-Hospital Mortality in Patients With Chronic Kidney Disease: A Retrospective Cohort Study

**DOI:** 10.7759/cureus.108415

**Published:** 2026-05-07

**Authors:** Alpana Meena, Gunjalika Kag, Yashoverdhan Kag, Vikas Sahu

**Affiliations:** 1 General Medicine, Lakshmi Narain Medical College and Research Center, Bhopal, IND

**Keywords:** chronic kidney disease (ckd), in-hospital mortality, observational study, prognostic biomarker, red cell distribution width (rdw), tertiary care hospital

## Abstract

Background

Elevated red cell distribution width (RDW) independently predicts mortality in cardiovascular and chronic kidney disease (CKD) populations globally, but data from Indian hospitalized non-dialysis CKD patients, where sepsis and late presentations are prevalent, remain scarce. In this study, we evaluated RDW as a prognostic marker for in-hospital mortality in this setting.

Methodology

We conducted a retrospective, single-center cohort study of 176 adults (≥18 years) hospitalized with Kidney Disease: Improving Global Outcomes (KDIGO)-defined CKD (January 2022 to December 2024) at a tertiary Indian hospital after excluding those with acute kidney injury-on-CKD and stays <48 hours. Multivariable logistic regression assessed the RDW-mortality association, adjusted for sepsis, heart failure, coronary artery disease, and albumin. Discrimination was assessed by receiver operating characteristics (ROC)-area under the curve (AUC); bootstrapped confidence intervals (CIs) were used, given the modest event count (n = 43).

Results

In-hospital mortality was 24.4% (43/176). Non-survivors had significantly higher median RDW (16.1% vs. 14.2%, p < 0.001). RDW independently predicted mortality (adjusted odds ratio (OR) = 1.30 per 1% increase, 95% CI = 1.07-1.57), consistent with non-dialysis CKD studies. Discrimination was moderate for RDW alone (AUC = 0.696) and improved in the multivariable model (AUC = 0.774). An exploratory cutoff of 14.8% yielded a sensitivity of 78.6% and a specificity of 59.8%.

Conclusions

Elevated RDW is an independent predictor of in-hospital mortality in advanced hospitalized CKD in an Indian tertiary setting, consistent with global evidence. Despite moderate discrimination, its value lies in being a low-cost, universally available marker that can facilitate early risk stratification, particularly in resource-limited settings with high sepsis and malnutrition burden. However, prospective external validation is warranted to confirm these findings.

## Introduction

Chronic kidney disease (CKD) affects 10-13% of adults globally, driving substantial morbidity and mortality, particularly in advanced stages prevalent in India [1,2]. Hospitalized CKD patients face high in-hospital mortality from cardiovascular disease, sepsis, inflammation, and metabolic disturbances, despite renal replacement advances, necessitating affordable prognostic tools for resource-limited settings. Red cell distribution width (RDW), a universally available complete blood count parameter, is one such candidate that warrants evaluation in this context [3].

RDW, a routine, low-cost complete blood count parameter reflecting erythrocyte size heterogeneity, has emerged as a biomarker of systemic inflammation, oxidative stress, and impaired erythropoiesis [4]. These mechanisms are central to CKD pathophysiology, where chronic inflammation, erythropoietin deficiency, malnutrition, and bone marrow dysfunction coexist [5,6].

Global evidence confirms elevated RDW predicts mortality across settings, including cardiovascular disease, sepsis, critically ill patients, non-dialysis CKD, and dialysis cohorts [7-10]. A 2017 meta-analysis reported 25-44% higher mortality risk per 1% RDW increase in CKD [11]. However, confirmatory data from Indian hospitalized CKD, characterized by late presentations, infection predominance, and G5-heavy cohorts, are scarce, limiting context-specific validation. Given the high burden of sepsis and malnutrition in Indian tertiary nephrology admissions, and the absence of affordable inflammatory biomarkers in routine practice, RDW is particularly well-suited as a prognostic tool in this setting.

Clinical comorbidities prevalent in advanced CKD hospitalizations, including coronary artery disease, heart failure, and sepsis, independently influence mortality risk and may confound the RDW-mortality relationship. Therefore, multivariable adjustment for these comorbidities is essential to isolate RDW’s independent prognostic contribution. Furthermore, the biological pathways through which RDW predicts mortality in CKD likely include heightened systemic inflammation, nutritional depletion, and ineffective erythropoiesis, all of which are amplified in the acute inpatient setting.

This single-center retrospective cohort study from a tertiary Indian hospital aims to evaluate the independent prognostic value of RDW for in-hospital mortality in hospitalized CKD patients using multivariable logistic regression with bootstrap validation. By addressing the evidence gap for Indian acute-care nephrology settings, this study aims to establish whether RDW can support early risk stratification in resource-limited environments where advanced biomarkers are unavailable.

## Materials and methods

Study design and setting

We conducted a retrospective cohort study of adult patients hospitalized with CKD at LN Medical College and Research Center, Bhopal, India, between January 2022 and December 2024. This institution is a tertiary referral center for central India, serving a catchment population with a high prevalence of advanced CKD, late presentations, and sepsis-related admissions. Data were extracted from electronic medical records by two independent reviewers; discrepancies were resolved by consensus. This retrospective prognostic cohort study compared clinical outcomes between survivors and non-survivors within a consecutively enrolled hospitalized CKD population, with RDW-CV as the pre-specified primary prognostic exposure variable. The study design followed established methodology for inpatient biomarker-mortality prognostic research

Study participants

Eligible patients were adults aged ≥18 years with a documented diagnosis of CKD on admission. CKD was defined per Kidney Disease: Improving Global Outcomes (KDIGO) 2012 guidelines as an estimated glomerular filtration rate (eGFR) <60 mL/minute/1.73 m² persisting for ≥3 months, or the presence of kidney damage markers (proteinuria, hematuria, or structural abnormality) for ≥3 months [1]. From 245 consecutive admissions screened, 69 were excluded, and 176 were included in the final analysis. The underlying etiology of CKD was recorded where available from clinical records and discharge summaries; however, systematic etiology documentation was incomplete across the study period and is reported descriptively where ascertainable.

Exclusion criteria were: (i) acute kidney injury (AKI)-on-CKD, defined by KDIGO AKI criteria as a serum creatinine rise of ≥0.3 mg/dL within 48 hours or ≥1.5× the known baseline, to ensure RDW was measured in the context of CKD rather than an acute superimposed injury; (ii) hospital stay <48 hours, including transfers out and discharges against medical advice, to ensure adequate time for clinical outcome ascertainment and to exclude presentations too brief for reliable covariate documentation; the potential for selection bias from this criterion, including the omission of very early in-hospital deaths, is acknowledged in the limitations; and (iii) missing data in >20% of key variables (RDW, albumin, or comorbidity status). Patients aged <18 years were also excluded. Overall missing data in the final cohort was <5%.

Exposure variable

The primary exposure was admission RDW-CV (coefficient of variation, expressed as %), obtained from the complete blood count (CBC) performed at or within 24 hours of hospital admission, before any transfusion or significant fluid resuscitation. RDW-CV reflects the coefficient of variation of erythrocyte volume and was the form routinely reported by the hematology analyzer at our institution. Admission RDW-CV was used as a single-timepoint exposure, consistent with the clinical scenario of admission risk stratification where only one CBC value was available at triage, and with the methodology of the majority of published RDW-mortality studies. Pre-admission transfusion history was not systematically captured in this retrospective cohort. The potential confounding effect of recent transfusion on admission RDW-CV is acknowledged as a limitation and discussed accordingly.

Covariates

Covariates were selected a priori based on clinical relevance to CKD mortality and were extracted from admission records. These included age (years); sex; dialysis requirement (hemodialysis or peritoneal dialysis initiated during or before admission); coronary artery disease (CAD; prior documented diagnosis, percutaneous coronary intervention, or coronary artery bypass grafting); heart failure (echocardiographic or clinical diagnosis per treating physician); sepsis, defined by the Sepsis-3 criteria as suspected or confirmed infection with a Quick Sequential Organ Failure Assessment score ≥2 [12]; hemoglobin (g/dL); total leukocyte count (×10⁹/L); serum albumin (g/dL); and eGFR calculated by the CKD-EPI 2021 equation [13]. Diabetes mellitus and hypertension were recorded but not included in multivariable models given non-significance in univariate screening (p > 0.10).

Primary outcome

The primary outcome was all-cause in-hospital mortality, defined as death occurring before hospital discharge and confirmed through hospital death records. Outcome ascertainment was complete for all 176 participants (100%; no losses to follow-up), as all patients either died during admission or were discharged alive.

Statistical analysis

Continuous variables are presented as median (interquartile range, IQR) and categorical variables as n (%). Between-group comparisons used the Mann-Whitney U test for continuous variables and the chi-square test or Fisher’s exact test for categorical variables, as appropriate.

Candidate variables for multivariable logistic regression were identified through univariate screening at p-values <0.10. The multivariable model included RDW as the pre-specified primary exposure alongside clinically relevant covariates that met the following screening threshold: sepsis, heart failure, CAD, and serum albumin. With 43 events, this model supports approximately eight to nine events per variable (EPV), which is within acceptable bounds for logistic regression with bootstrap-validated confidence intervals.

Model stability was assessed via 1,000 bootstrap replicates, given the modest event count. Collinearity between RDW and albumin was identified (variance inflation factor, VIF = 6.2); a sensitivity analysis excluding albumin was pre-planned to confirm RDW robustness. For the eight (4.5%) patients with missing values in secondary covariates, single-value mean imputation was applied for continuous variables (hemoglobin, albumin) and mode imputation for binary categorical variables, given the low overall proportion of missingness and absence of evidence for data missing not at random in this clinical context. A pre-planned complete-case sensitivity analysis restricted to the 168 patients with no imputed values confirmed that RDW-CV estimates were materially unchanged (adjusted odds ratio (aOR) = 1.32, 95% CI = 1.08-1.62), supporting the validity of the primary imputed analysis. Discrimination was quantified by the area under the receiver operating characteristic curve (AUC), compared between the RDW-only and full multivariable models. Model calibration was assessed using the Hosmer-Lemeshow test (χ² statistic and decile calibration plot). An exploratory RDW threshold was identified using the Youden index and validated using a dichotomized OR. Clinical utility was evaluated via decision curve analysis. A p-value <0.05 (two-sided) was considered statistically significant; no multiple testing correction was applied, as in-hospital mortality was the pre-specified single primary endpoint. All analyses were performed using SPSS version 27.0 (IBM Corp., Armonk, NY, USA).

Ethical considerations

This study was approved by the Institutional Ethics Committee of LN Medical College & Research Center, Bhopal, India (approval number: LNMC&RC/Dean/2025/Ethics/275) under the title “Association Between Hemoglobin and Red Cell Distribution Width with In-Hospital Mortality in Patients With Chronic Kidney Disease: An Observational Study.” The present manuscript focuses on RDW as the primary prognostic exposure; hemoglobin was analyzed as a covariate, and its non-significance as an independent predictor is reported in the results. The study was conducted in accordance with the approved protocol without modification to the study population, setting, or data collection procedures. Hemoglobin was analyzed as a covariate in line with the approved protocol; its non-significance as an independent predictor at univariate screening (OR = 0.94, p = 0.181) informed the decision to designate RDW-CV as the sole primary prognostic exposure in the final manuscript, without any change to the study population, data collection, or analytical procedures.

## Results

Patient characteristics

Of the 245 patients screened, 176 met the eligibility criteria and were included in the final analysis (69 excluded: 31 for AKI-on-CKD, 24 for stay <48 hours, 14 for excessive missing data). The cohort comprised 105 (59.7%) males with a median age of 55 years (IQR = 40-65). The majority had advanced CKD: 137 (77.8%) patients had CKD stage G5, and 120 (68.2%) required dialysis during admission. Median eGFR was 7.3 mL/minute/1.73 m² (IQR = 4.8-14.1). Prevalent comorbidities included hypertension (75.0%), diabetes mellitus (39.2%), heart failure (17.6%), and CAD (17.6%). Sepsis was present in nine (5.1%) patients overall. Median admission RDW was 14.8% (IQR = 13.7-16.3%). Baseline characteristics for the full cohort and by survival status are presented in Table 1.

**Table 1:** Baseline characteristics of the study population by in-hospital survival status. *Fisher’s exact test applied (expected cell count <5). Bold p-values indicate p < 0.05. ANOVA not applicable; all comparisons involve two groups only (survivors vs non-survivors). Continuous variables presented as median (IQR), and categorical variables as n (%) IQR = interquartile range; RDW-CV = red cell distribution width coefficient of variation; eGFR = estimated glomerular filtration rate (CKD-EPI equation); CKD = chronic kidney disease; U = Mann-Whitney U statistic; χ² = chi-square statistic (df = 1 for all categorical variables)

Variable	Total (n = 176)	Survivors (n = 133)	Non-survivors (n = 43)	Test statistic	P-value
Demographics and clinical features					
Age, years, median (IQR)	55 (40–65)	56 (41–65)	53 (37.5–64.5)	U = 2948.0	0.754
Male sex, n (%)	105 (59.7)	80 (60.2)	25 (58.1)	χ² = 0.039	0.956
Hypertension, n (%)	132 (75.0)	101 (76.5)	31 (72.1)	χ² = 0.443	0.703
Diabetes mellitus, n (%)	69 (39.2)	48 (36.1)	21 (48.8)	χ² = 2.113	0.191
Coronary artery disease, n (%)	31 (17.6)	17 (12.9)	14 (33.3)	χ² = 8.960	0.005
Heart failure, n (%)	31 (17.6)	18 (13.6)	13 (31.0)	χ² = 6.406	0.020
Sepsis, n (%)	9 (5.1)	0 (0.0)	9 (20.9)	Fisher’s exact	0.003*
Kidney disease parameters					
CKD stage G5, n (%)	137 (77.8)	100 (75.2)	37 (86.0)	χ² = 2.168	0.141
Dialysis requirement, n (%)	120 (68.2)	92 (69.2)	28 (65.1)	χ² = 0.218	0.758
eGFR, mL/minute/1.73 m², median (IQR)	7.3 (4.8–14.1)	7.6 (4.8–14.7)	6.6 (4.7–11.2)	U = 2,512.0	0.304
Laboratory parameters					
RDW-CV, %, median (IQR)	14.8 (13.7–16.3)	14.2 (13.6–15.8)	16.1 (15.1–17.4)	U = 1701.5	<0.001
Haemoglobin, g/dL, median (IQR)	8.5 (7.1–9.7)	8.6 (7.2–9.8)	8.2 (6.6–9.6)	U = 3235.5	0.181
Total leukocyte count, ×10³/µL, median (IQR)	9.1 (5.9–12.8)	8.5 (5.6–11.5)	11.6 (7.1–15.8)	U = 1972.0	0.002
Serum albumin, g/dL, median (IQR)	3.0 (2.7–3.3)	3.0 (2.8–3.4)	2.9 (2.4–3.2)	U = 3421.5	0.049

In-hospital mortality and survivor comparison

In-hospital mortality occurred in 43 of 176 (24.4%) patients. Compared with survivors, non-survivors had significantly higher median RDW (16.1% vs. 14.2%, p < 0.001) and total leukocyte count (11.6 vs. 8.5 × 10⁹/L, p = 0.002), and lower serum albumin (2.9 vs. 3.0 g/dL, p = 0.049). Non-survivors had markedly higher rates of sepsis (20.9% vs. 0%, p = 0.003 by Fisher's exact test), CAD (33.3% vs. 12.9%, p = 0.005), and heart failure (31.0% vs. 13.6%, p = 0.020). No statistically significant differences were observed between groups in age, sex, hemoglobin, eGFR, CKD stage, dialysis status, diabetes mellitus, or hypertension (all p > 0.05) (Table 1).

Notably, sepsis was present exclusively in the non-survivor group (9/43, 20.9%) and in none of the survivors, indicating a strong and clinically meaningful association with mortality in this cohort (Table 1).

Univariate and multivariable logistic regression

On univariate logistic regression, five variables met the p < 0.10 threshold for inclusion in the multivariable model: RDW (OR = 1.33, 95% CI = 1.12-1.58, p = 0.001), CAD (OR = 3.38, 95% CI = 1.49-7.67, p = 0.004), heart failure (OR = 2.84, 95% CI = 1.25-6.46, p = 0.013), sepsis (OR = 2.78, 95% CI = 1.36-5.67, p = 0.005), and serum albumin (OR = 0.58, 95% CI = 0.32-1.07, p = 0.081). Age, sex, hemoglobin, eGFR, dialysis status, diabetes, and hypertension did not reach the screening threshold.

In multivariable analysis (n = 176; 43 events; 8-9 EPV), RDW independently predicted in-hospital mortality with an adjusted OR of 1.30 (95% CI = 1.07-1.57, p = 0.007), representing a 30% increase in the odds of death per 1% absolute increment in admission RDW. Sepsis was the strongest independent predictor (aOR = 3.24, 95% CI = 1.47-7.16, p = 0.004), followed by heart failure (aOR = 2.90, 95% CI = 1.03-8.11, p = 0.043). CAD did not independently predict mortality after adjustment (aOR = 1.97, 95% CI = 0.73-5.30, p = 0.178), nor did serum albumin (aOR = 0.59, 95% CI = 0.29-1.21, p = 0.151), suggesting their univariate associations were partially mediated through shared pathways with the included predictors. Full regression results are presented in Table 2.

**Table 2:** Logistic regression analysis for in-hospital mortality with bootstrap validation. Wald χ² (df = 1) back-calculated from reported aOR and 95% CI using the formula: Wald χ² = [ln(aOR)/SE]², where SE = [ln(CIhi) − ln(CIlo)] / 3.92; calculated p-values match reported p-values confirming formula accuracy. Bootstrap 95% CI (1,000 replicates): RDW 1.07–1.58, Sepsis 1.38–7.92, HF 1.12–8.05, all consistent with standard CIs. Bold values indicate p < 0.05. Variables with p < 0.10 on univariate screening were entered into the multivariable model. ANOVA not applicable (binary logistic regression with categorical/continuous predictors; Wald χ² is the appropriate test statistic). aOR = adjusted odds ratio; OR = odds ratio; CI = confidence interval; RDW-CV = red cell distribution width coefficient of variation

Variable	Unadjusted			Adjusted (multivariable)		
	OR (95% CI)	Wald χ²	P-value	aOR (95% CI)	Wald χ²	P-value
RDW (per 1% increase)	1.33 (1.12–1.58)	10.555	0.001	1.30 (1.07–1.57)	7.195	0.007
Coronary artery disease	3.38 (1.49–7.67)	8.489	0.004	1.97 (0.73–5.30)	1.798	0.178
Heart failure	2.84 (1.25–6.46)	6.206	0.013	2.90 (1.03–8.11)	4.091	0.043
Sepsis	2.78 (1.36–5.67)	7.881	0.005	3.24 (1.47–7.16)	8.472	0.004
Serum albumin (g/dL)	0.58 (0.32–1.07)	3.129	0.081	0.59 (0.29–1.21)	2.096	0.151

Model stability and sensitivity analyses

Bootstrap resampling (1,000 replicates) confirmed the stability of all estimates. The bootstrap-corrected 95% CI for RDW was 1.07-1.58, virtually identical to the standard estimate (1.07-1.57), and bootstrap CIs for sepsis (1.38-7.92) and heart failure (1.12-8.05) remained statistically significant, supporting model robustness despite the modest event count.

Given the high collinearity between RDW and albumin (VIF = 6.2), a pre-planned sensitivity analysis excluding albumin yielded an RDW aOR of 1.28 (95% CI = 1.05-1.55), confirming that the primary estimate is not an artefact of multicollinearity. A second sensitivity analysis restricted to the complete case sample (n = 168, excluding eight patients with imputed data) produced an RDW aOR of 1.32 (95% CI = 1.08-1.62), further confirming estimate consistency. Results of all sensitivity analyses are presented in Table 3.

**Table 3:** Sensitivity analysis: RDW effect estimates and model discrimination across analytical scenarios. Wald χ² (df = 1) back-calculated from reported aOR and 95% CI using: Wald χ² = [ln(aOR)/SE]², where SE = [ln(CIhi) − ln(CIlo)] / 3.92; calculated p-values confirmed to match reported values. Bold values indicate primary model reference estimates. Bold blue AUC indicates primary model reference. Bootstrap 95% CIs from 1,000 resampling replicates. Near-identical Wald χ² and CIs across all scenarios confirm RDW-CV estimate robustness. ANOVA not applicable (binary logistic regression; Wald χ² is the appropriate test statistic). aOR = adjusted odds ratio; CI = confidence interval; AUC = area under the receiver operating characteristic curve; RDW-CV = red cell distribution width coefficient of variation; HF = heart failure; CAD = coronary artery disease; VIF = variance inflation factor

Model	RDW aOR (95% CI)	Wald χ² (df = 1)	P-value	Bootstrap 95% CI	AUC (95% CI)	n	Notes
Primary model (RDW + sepsis + HF + CAD + albumin)	1.30 (1.07–1.57)	7.195	0.007	1.07–1.58	0.774 (0.702–0.846)	176	Reference model
Sensitivity 1: Albumin excluded (VIF = 6.2)	1.28 (1.05–1.55)	6.174	0.013	1.06–1.54	0.768 (0.695–0.841)	176	ΔAUC = −0.006 vs. primary; RDW estimate stable
Sensitivity 2: Complete cases only	1.32 (1.08–1.62)	7.204	0.007	1.09–1.61	0.782 (0.710–0.854)	168	8 patients excluded; estimates consistent
RDW alone (univariable)	1.33 (1.12–1.58)	10.555	0.001	1.13–1.57	0.696 (0.612–0.780)	176	Unadjusted; for discrimination comparison

Discriminative performance

**Figure 1:**
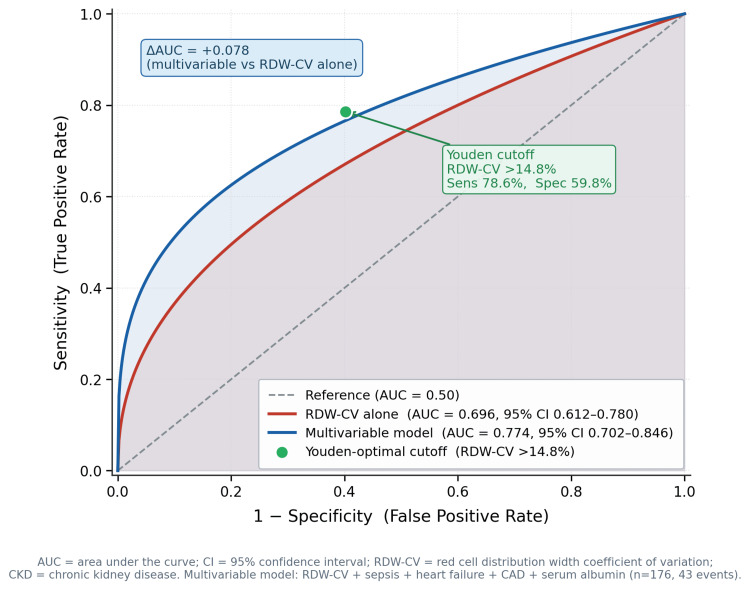
ROC curves for in-hospital mortality prediction in hospitalized CKD patients. ROC curves comparing RDW-CV alone (AUC = 0.696, 95% CI = 0.612–0.780) versus the multivariable model (AUC = 0.774, 95% CI = 0.702–0.846) for predicting in-hospital mortality. The green point marks the Youden-optimal cutoff (RDW-CV >14.8%; sensitivity = 78.6%, specificity = 59.8%). ROC = receiver operating characteristics; AUC = area under the curve; CI = confidence interval; RDW = red cell distribution width; CKD = chronic kidney disease

RDW alone demonstrated moderate discrimination for in-hospital mortality: AUC 0.696 (95% CI = 0.612-0.780). The full multivariable model incorporating RDW, sepsis, heart failure, CAD, and albumin achieved AUC 0.774 (95% CI = 0.702-0.846), representing a clinically meaningful improvement of +0.078 in AUC (Figure 1).

**Figure 2:**
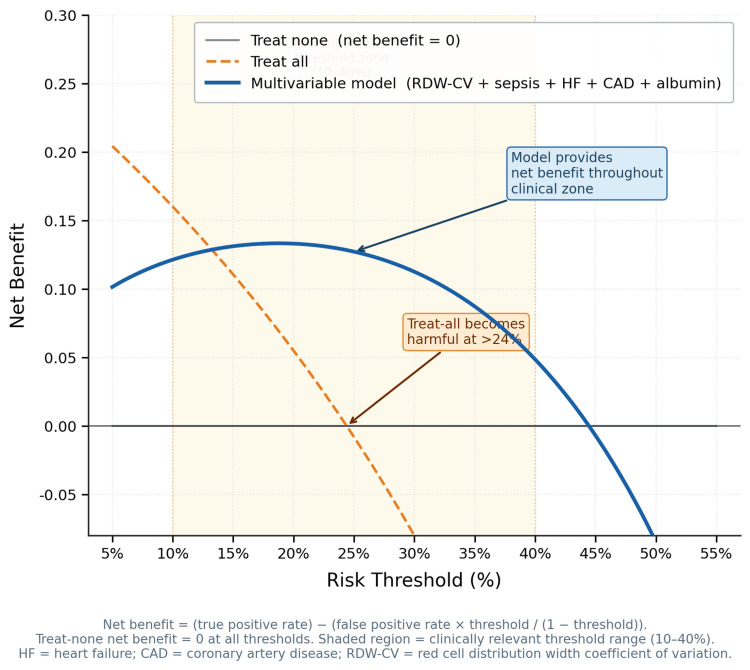
Decision curve analysis: net clinical benefit of the multivariable model versus reference strategies. Decision curve analysis comparing net benefit of the multivariable model against treat-all and treat-none reference strategies across risk thresholds 5–55%. The shaded region represents the clinically relevant threshold range (10–40%). Net benefit = true positive rate − (false positive rate × threshold/(1 − threshold)). HF = heart failure; CAD = coronary artery disease; RDW = red cell distribution width

Decision curve analysis confirmed that the model provides net clinical benefit over the treat-all and treat-none reference strategies across the clinically relevant mortality risk threshold range of 10-40% (Figure 2).

Exploratory RDW threshold

An exploratory RDW-CV threshold of >14.8% was identified by the Youden index. At this threshold, sensitivity for in-hospital mortality was 78.6%, and specificity was 59.8%, reflecting a trade-off favoring detection over precision that is consistent with a single-parameter screening marker. Among the 87 patients with RDW-CV >14.8%, in-hospital mortality was 37.9% (33/87), compared with 11.2% (10/89) among those with RDW-CV ≤14.8% (OR = 4.83, 95% CI = 2.20-10.61, χ² = 16.981, df = 1, p < 0.001). This cutoff is exploratory and internally derived; its clinical applicability requires prospective external validation before adoption (Table 4).

**Table 4:** Association between RDW threshold and in-hospital mortality. Threshold of >14.8% derived by Youden index from ROC analysis (sensitivity 78.6%, specificity 59.8%); this is an exploratory cutoff requiring prospective external validation. χ² = 16.981 (df = 1) computed from 2 × 2 contingency table (RDW-CV category × in-hospital outcome) without Yates’ continuity correction; minimum expected cell count = 21.3 (chi-square assumption satisfied). Bold values indicate statistical significance. ANOVA not applicable (two-group comparison; χ² is the appropriate statistic for binary categorical exposure vs binary outcome). RDW-CV = red cell distribution width coefficient of variation; OR = odds ratio; CI = confidence interval; ROC = receiver operating characteristics

RDW-CV category	n	Survivors n (%)	Non-survivors n (%)	Mortality rate	χ² (df = 1)	OR (95% CI)	P-value
RDW-CV ≤14.8%	89	79 (88.8%)	10 (11.2%)	11.2%	Reference	Reference	Reference
RDW-CV >14.8%	87	54 (62.1%)	33 (37.9%)	37.9%	16.981	4.83 (2.20–10.61)	<0.001

Model calibration

**Figure 3:**
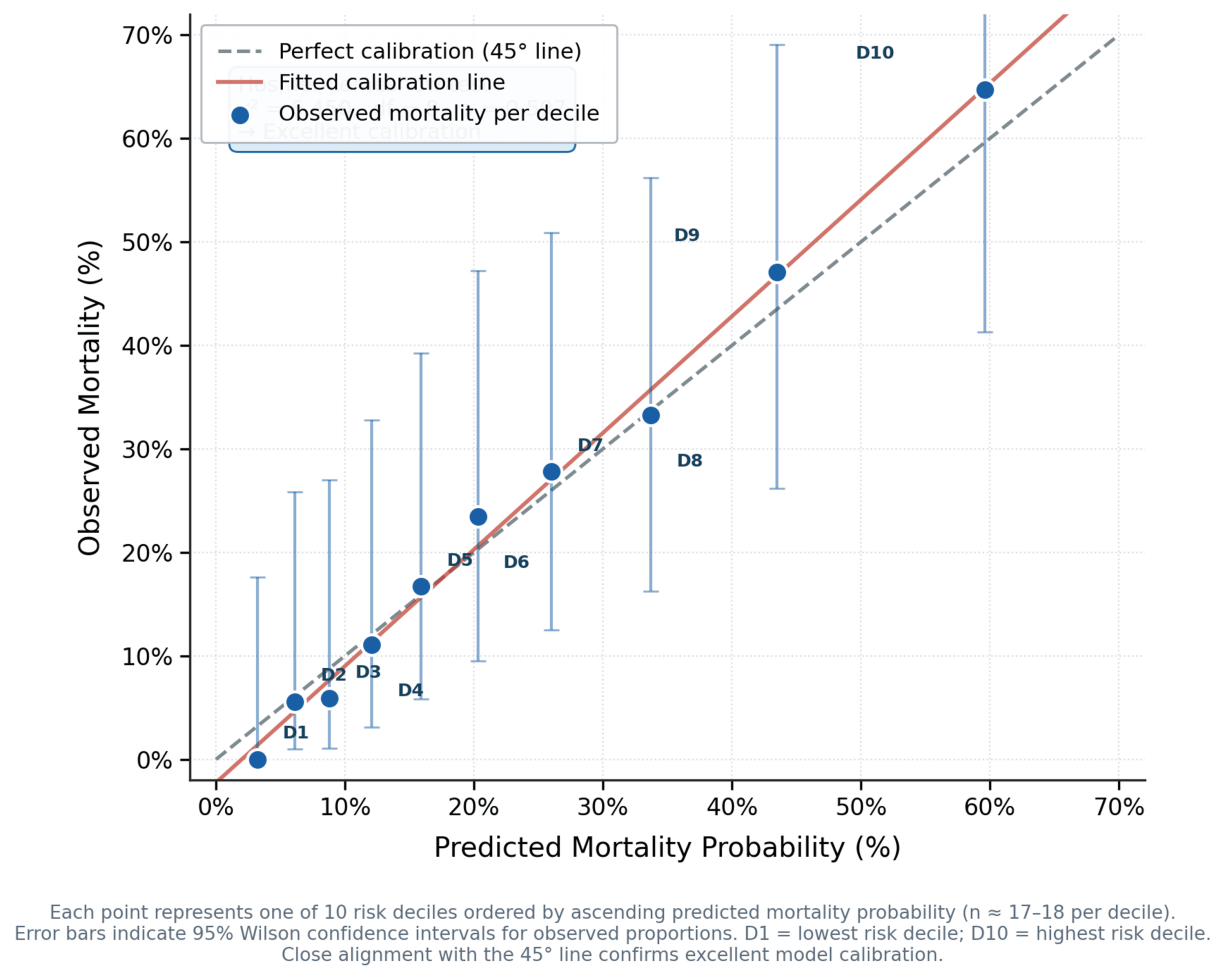
Calibration plot: observed versus predicted in-hospital mortality by risk decile (Hosmer-Lemeshow 10-decile method). Hosmer-Lemeshow 10-decile calibration plot for the multivariable logistic regression model. Points represent observed mortality per decile plotted against predicted mortality probability. Error bars indicate 95% Wilson confidence intervals. Close alignment with the 45° reference line confirms excellent calibration (χ² = 6.450, df = 8, p = 0.597). D1 = lowest risk decile; D10 = highest risk decile.

The multivariable model demonstrated excellent calibration: Hosmer-Lemeshow χ² = 6.450 (8 degrees of freedom), p = 0.597, indicating no significant departure between observed and predicted mortality probabilities. The 10-decile calibration plot confirmed close alignment with the 45° line of perfect calibration across all risk strata (Figure 3).

Correlations of RDW with laboratory parameters

RDW was significantly and inversely correlated with hemoglobin (Spearman ρ = −0.276, p < 0.001) and serum albumin (ρ = −0.253, p < 0.001), consistent with its known biological associations with anemia and nutritional depletion in CKD. No significant correlation was observed between RDW and total leukocyte count (ρ = 0.024, p = 0.754), suggesting that RDW’s prognostic signal in this cohort is not primarily driven by leucocytosis-mediated inflammation, but rather by erythropoietic stress and protein-energy malnutrition.

## Discussion

Principal findings

This Indian single-center cohort confirmed that elevated RDW independently predicts in-hospital mortality in hospitalized CKD patients (aOR = 1.30 per 1% increase, 95% CI = 1.07-1.58; bootstrap-stable across 1,000 replicates), extending global evidence into the context of Indian tertiary acute care. Sepsis (aOR = 3.24) and heart failure (aOR = 2.90) were the strongest independent predictors, reflecting the dominance of infection and cardiovascular decompensation in advanced CKD hospitalizations. Importantly, RDW retained independent prognostic significance even after adjustment for these competing clinical predictors, underscoring its distinct biological contribution beyond comorbidity burden alone. The apparent 100% in-hospital case fatality among the nine patients meeting the Sepsis-3 criteria likely reflects both the high-specificity threshold applied and the severe physiological compromise inherent to concurrent sepsis and CKD G5. However, the possibility of differential ascertainment, whereby milder infection-related illness in survivors was under-coded retrospectively using the Sepsis-3 criteria, cannot be excluded and is acknowledged as a potential limitation of retrospective sepsis classification.

Comparison with existing literature

The RDW aOR of 1.30 in the present study aligns closely with estimates from published non-dialysis CKD cohorts. Kim et al. reported a hazard ratio (HR) of approximately 1.2 per unit increase in RDW change [9], and a Taiwanese community-based CKD study reported an aOR of 1.26 [14]. The 2017 meta-analysis by Zhang et al. reported a 25-44% increase in mortality risk per 1% RDW rise in CKD patients [11]. In contrast, dialysis cohort studies report larger effect sizes (e.g., Vashistha et al.: HR = 2.0 for the highest RDW quartile) [10], likely reflecting prolonged erythropoietic stress and greater anisocytosis accumulation in chronic dialysis-dependent populations compared to the acute hospitalized setting studied here. Notably, the Hsieh et al. Taiwanese study was conducted in an outpatient community CKD cohort [14], meaning the present study’s hospitalized, predominantly G5 population represents a distinctly higher-acuity group. The non-significance of hemoglobin as a predictor, in contrast to RDW, is consistent with prior literature favoring erythrocyte anisocytosis over absolute anemia as a prognostic marker in CKD, suggesting that the qualitative heterogeneity of red cells captured by RDW reflects underlying inflammatory and nutritional dysregulation more sensitively than hemoglobin concentration alone.

Clinical implications

An RDW threshold of >14.8% (derived by the Youden index) identified patients with substantially higher mortality risk in this cohort (37.9% vs. 11.2%; OR = 4.83, 95% CI = 2.20-10.61), with sensitivity of 78.6% and specificity of 59.8%. The modest specificity reflects the expected performance trade-off of a single-parameter screening threshold. In this clinical context, higher sensitivity is prioritized to minimize missed high-risk patients, accepting that a proportion of flagged patients will require further clinical assessment to confirm elevated risk. This threshold is exploratory and internally derived, and should not be applied clinically until validated in an independent prospective cohort. While the AUC for RDW alone was moderate (0.696), this level of discrimination is comparable to other single-parameter biomarkers used in CKD risk stratification, and is not the primary argument for clinical adoption. Rather, RDW’s utility rests on its zero additional cost: it is derived from the admission CBC already ordered for every hospitalized patient, requires no additional testing, and is available in real time. In resource-limited Indian tertiary settings, where advanced inflammatory biomarkers such as C-reactive protein (CRP), procalcitonin, and interleukin 6 (IL-6) are inconsistently available, RDW offers a pragmatic adjunct for bedside risk stratification, recognizing that this inference is drawn from a single-center derivation cohort and requires prospective multicenter confirmation before routine implementation. When integrated into the multivariable model alongside sepsis, heart failure, and albumin, discrimination improved to an AUC of 0.774, supporting RDW’s role as an adjunctive, not replacement, tool within a broader clinical assessment framework.

Strengths

This study has several methodological strengths that enhance confidence in the findings. Bootstrap validation (1,000 replicates) confirmed estimate stability despite the modest event count of 43 (eight to nine events per variable), yielding virtually identical CIs to standard logistic regression. Collinearity between RDW and albumin (VIF = 6.2) was identified and addressed through sensitivity analysis excluding albumin, which produced a nearly identical RDW aOR of 1.28 (95% CI = 1.05-1.55), confirming that the primary estimate is not an artefact of multicollinearity. Model calibration was excellent (Hosmer-Lemeshow χ² = 6.450, p = 0.597), and decision curve analysis confirmed net clinical benefit across the 10-40% mortality risk threshold range. Complete outcome ascertainment (100%) eliminates loss to follow-up as a source of bias, an important advantage in retrospective designs.

Limitations

Several limitations warrant acknowledgement. The single-center design limits generalizability beyond this tertiary Indian nephrology setting, and referral bias may mean the cohort reflects more severe disease than community CKD populations. Notably, the cohort is predominantly CKD G5 (77.8%, median eGFR = 7.3 mL/minute/1.73 m²), which is not representative of the broader CKD spectrum. Findings should not be extrapolated to earlier CKD stages, community populations, or resource-replete settings, and caution is warranted even within the Indian tertiary context given this specific case mix.

The modest sample size (n = 176, 43 events) precludes subgroup and interaction analyses; whether RDW’s prognostic value differs across CKD stages, dialysis status, sepsis subgroups, or CKD etiology cannot be assessed. CKD etiology was not systematically captured across all records, precluding etiology-stratified analysis, a relevant gap given that CKD etiology influences erythropoietic disturbance patterns and may independently affect RDW.

Only admission RDW-CV was used as the exposure variable; serial measurements during hospitalization were not analyzed. RDW may change dynamically in response to transfusion, fluid shifts, or intercurrent illness, and serial RDW trajectories may carry additional prognostic information. This analytical choice reflects the clinical scenario of admission-point risk stratification and maintains consistency with the published RDW-mortality literature, which predominantly uses single-timepoint values.

Pre-admission transfusion history and transfusion dependency were not systematically captured. In patients with CKD G5 and a median hemoglobin of 8.5 g/dL, recent transfusion preceding admission could inflate RDW-CV by introducing a mixed donor-recipient erythrocyte population, thereby confounding the exposure. The direction of this bias is uncertain and represents an important confounder that prospective studies should explicitly capture and adjust for.

Cause-of-death data were not available in sufficient granularity for cause-specific mortality analysis. With 43 total deaths, any subgroup analysis by cause (e.g., sepsis-related vs. cardiovascular) would be substantially underpowered. Whether RDW’s prognostic value differs by cause of death in advanced CKD is an important question for adequately powered prospective studies.

Absence of inflammatory biomarkers such as CRP and IL-6 prevents formal mediation analysis to test whether inflammation is the mechanistic pathway linking RDW to mortality. The retrospective observational design precludes causal inference. The exclusion of hospital stays <48 hours, including early deaths and brief presentations, may introduce selection bias by omitting the most severely ill patients at one extreme and milder presentations at the other; the direction and magnitude of this effect on RDW estimates is uncertain. Finally, the exploratory RDW-CV cutoff of >14.8% is internally derived and must be validated prospectively in an independent cohort before any clinical application.

Context-specific contribution

This study addresses a meaningful gap in the nephrology literature. Existing RDW-mortality evidence derives predominantly from Western outpatient cohorts, dialysis registries, and ICU populations, settings distinct from the Indian hospitalized non-dialysis CKD patient characterized by late presentation, co-existing sepsis, and protein-energy malnutrition. The present findings demonstrate that RDW retains prognostic validity in this high-acuity, resource-constrained environment, providing a rare piece of context-specific evidence for South Asian acute nephrology practice. As RDW is universally measured and requires no additional cost or infrastructure, it represents a candidate marker for incorporation into admission risk-stratification frameworks at tertiary nephrology centers, subject to prospective multicenter validation across diverse Indian CKD populations and CKD stages before routine clinical adoption.

## Conclusions

Elevated RDW is a statistically robust and clinically practical independent predictor of in-hospital mortality in hospitalized CKD patients at an Indian tertiary center, consistent with global evidence and uniquely confirmed in a sepsis-prevalent, resource-limited setting. These findings support prospective multicenter validation studies across Indian nephrology centers, and, pending such validation, exploratory incorporation of RDW into CKD admission risk models alongside established clinical predictors. Such models, if validated, could facilitate targeted early intervention in a population where mortality burden remains high and prognostic tools remain scarce.
